# Simplified post processing of cine DENSE cardiovascular magnetic resonance for quantification of cardiac mechanics

**DOI:** 10.1186/s12968-014-0094-9

**Published:** 2014-11-28

**Authors:** Jonathan D Suever, Gregory J Wehner, Christopher M Haggerty, Linyuan Jing, Sean M Hamlet, Cassi M Binkley, Sage P Kramer, Andrea C Mattingly, David K Powell, Kenneth C Bilchick, Frederick H Epstein, Brandon K Fornwalt

**Affiliations:** Department of Pediatrics and Saha Cardiovascular Research Center, University of Kentucky, Lexington, KY USA; Department of Biomedical Engineering, University of Kentucky, Lexington, KY USA; Department of Electrical Engineering, University of Kentucky, Lexington, KY USA; Department of Medicine, University of Virginia, Charlottesville, VA USA; Department of Biomedical Engineering, University of Virginia, Charlottesville, VA USA

**Keywords:** DENSE, Cardiovascular magnetic resonance, Cardiac mechanics

## Abstract

**Background:**

Cardiovascular magnetic resonance using displacement encoding with stimulated echoes (DENSE) is capable of assessing advanced measures of cardiac mechanics such as strain and torsion. A potential hurdle to widespread clinical adoption of DENSE is the time required to manually segment the myocardium during post-processing of the images. To overcome this hurdle, we proposed a radical approach in which *only* three contours per image slice are required for post-processing (instead of the typical 30–40 contours per image slice). We hypothesized that peak left ventricular circumferential, longitudinal and radial strains and torsion could be accurately quantified using this simplified analysis.

**Methods and Results:**

We tested our hypothesis on a large multi-institutional dataset consisting of 541 DENSE image slices from 135 mice and 234 DENSE image slices from 62 humans. We compared measures of cardiac mechanics derived from the simplified post-processing to those derived from original post-processing utilizing the full set of 30–40 manually-defined contours per image slice. Accuracy was assessed with Bland-Altman limits of agreement and summarized with a modified coefficient of variation. The simplified technique showed high accuracy with all coefficients of variation less than 10% in humans and 6% in mice. The accuracy of the simplified technique was also superior to two previously published semi-automated analysis techniques for DENSE post-processing.

**Conclusions:**

Accurate measures of cardiac mechanics can be derived from DENSE cardiac magnetic resonance in both humans and mice using a simplified technique to reduce post-processing time by approximately 94%. These findings demonstrate that quantifying cardiac mechanics from DENSE data is simple enough to be integrated into the clinical workflow.

## Background

Advancements in cardiovascular magnetic resonance (CMR) have made it possible to accurately assess not only standard clinical metrics such as ejection fraction (EF) and ventricular volumes, but also advanced measures of cardiac mechanics such as strain and torsion [1-3]. These measures of cardiac mechanics demonstrate a higher prognostic value when combined with both clinical risk factors and more traditional metrics such as ejection fraction [4]. Cine displacement encoding with stimulated echoes (DENSE) is an advanced CMR technique for measuring cardiac mechanics which encodes the displacement of the myocardium into the phase of the MR signal [5]. DENSE data can be used to reproducibly quantify cardiac mechanics, ventricular volumes, and ejection fraction [6-8]. Recent work has demonstrated the clinical utility of DENSE, specifically for predicting response to cardiac resynchronization therapy [9]. Despite its apparent clinical utility, DENSE has yet to see widespread adoption in clinical practice.

One of the primary hurdles for clinical adoption of advanced CMR techniques, including DENSE, is the amount of post-processing required to condense hundreds of images down to several useful metrics. The majority of CMR analyses require that an experienced observer manually delineate the endocardium and epicardium of the heart within each image. When the user is only required to define the end systolic and diastolic images for calculation of EF and volumes, the workload is manageable; however, advanced analysis often requires cine acquisitions with boundaries delineated on 20–30 image frames per heartbeat. The recent surge in three-dimensional and high-resolution imaging techniques has further increased post-processing time. This time required for manual post-processing greatly limits the clinical use of advanced CMR techniques.

In an attempt to overcome this limitation, many techniques have been developed to segment the myocardium [10-13]. Over time these techniques have become more complex and theoretically more accurate, but clinically, agreement between two *segmentations* of the ventricle is irrelevant *if* they provide the *same measures of cardiac function*. Compared to traditional methods in which myocardial contours are used directly to derive cardiac mechanics, DENSE analysis relies upon the underlying displacement data encoded in the images, and the contours are used solely to mask out irrelevant regions. Due to this fundamental difference, we hypothesized that accurate measures of cardiac mechanics can be obtained from DENSE images in a fraction of the time compared to current standards by using a simplified approach. Our proposed “simplified” post-processing utilizes only three contours (which are the same contours drawn during clinical workflow for calculation of left ventricular mass, volumes and ejection fraction [8]): the endocardial and epicardial end-diastolic contours and the endocardial end-systolic contour. We tested our hypothesis using a large, multi-institutional dataset consisting of 541 DENSE image slices acquired from 135 mice and 234 DENSE image slices from 62 humans.

## Methods

### Subjects

The data and subjects selected for this study were intended to represent a heterogeneous population to emphasize the general applicability of the findings. We therefore included data acquired at both the University of Kentucky and the University of Virginia consisting of: 1) 541 separate mouse DENSE imaging slices from 67 healthy controls and 68 obese mice with cardiac remodeling and dysfunction 2) 60 DENSE imaging slices from 12 healthy human subjects and 3) 174 DENSE imaging slices from 50 patients with heart failure. All animal procedures conformed to the Public Health Service policies for humane care and use of animals, and all procedures were approved by the institutional animal care and use committee at our Institution. All human subjects gave informed consent and protocols were approved by our Institutional Review Board.

### Image acquisition

All mouse imaging was performed on a 7 T Bruker ClinScan system (Bruker, Ettlingen, Germany) equipped with a 4-element phased array cardiac coil. Short-axis images (basal, mid-ventricular, apical) and long-axis images (two and four-chamber) were acquired with 13–20 frames per cardiac cycle. In-plane displacements were encoded for each image. Acquisition parameters were: TR = 7.4 ms, TE = 1.0 ms, Acquisition Matrix = 128 × 128, Pixel Spacing = 0.25 mm, Slice Thickness = 1 mm, Flip Angle = 20°, 36 inter-leaved spirals, displacement encoding frequency (k_e_) =1.0 cycles/mm.

Human subject imaging was performed on either a 3 T Siemens Tim Trio or 1.5 T Siemens Avanto system (Siemens Healthcare, Erlangen, Germany) with a 6-element phased array cardiac coil and a 24-element spine coil. Short-axis images (basal, mid-ventricular, apical) and long-axis images (two and four-chamber) were acquired with 17–28 frames per cardiac cycle. In-plane displacements were encoded for each image. Acquisition parameters were: TR = 17 ms, TE = 1.1 ms, Acquisition Matrix = 128 × 128, Pixel Spacing = 2.6 – 2.8 mm, Slice Thickness = 8 mm, Flip Angle = 20°, 6 inter-leaved spirals, k_e_ = 0.1 cycles/mm.

### DENSE post-processing

**Figure 1:**
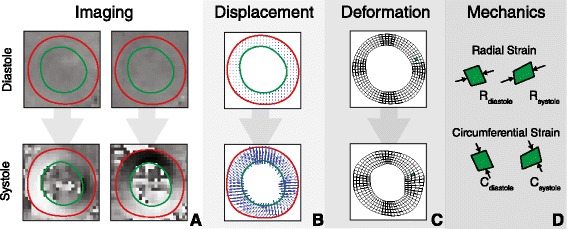
**Cine DENSE analysis.** Tissue displacements are encoded into the phase of the signal **(A)** and using endocardial and epicardial boundaries (green and red, respectively), it is possible to derive a displacement field **(B)**. This displacement field is then used to deform a mesh of the resting configuration **(C)** from which we can derive regional cardiac mechanics such as strain **(D)**.

All DENSE data was processed using a standard protocol described previously [14]. Briefly, black-blood magnitude images and displacement-encoded images for each spatial direction were imported from the MR scanner (Figure 1A). An experienced observer annotated the endocardial and epicardial boundaries of the left ventricle in each imaging slice at all acquired time frames. Boundaries from an end-diastolic frame (termed the “resting configuration”) (Figure 1C, top) were used to generate a two-dimensional mesh representation of the myocardium (with 128 elements circumferentially and 5 elements radially). Contours on all other frames were used to indicate which pixels represent actual displacements of the myocardium and to exclude pixels which fall outside of the myocardium and consist solely of phase noise (Figure 1A, red and green contours). The phase data lying between these contours was then unwrapped using a path following algorithm guided by manual selection of several seed points indicating unwrapped phase pixels [15]. Two dimensional displacement vectors were then computed and the temporal trajectory of each data point was smoothed with a 10^th^ order polynomial (Figure 1B). The mesh of the resting configuration was deformed using the computed displacement field for each acquisition frame (Figure 1C, bottom). Using this deformation and the user-defined anterior insertion of the right ventricle, the circumferential and radial components of the 2D Lagrangian strains were computed for each element of the mesh and expressed as a percentage (Figure 1D). Identical post-processing was performed on both short and long-axis images. Torsion was calculated as the slope of the linear regression line between twist angle and image slice location.

### Creation of simplified boundaries

To compare our new simplified approach to the original method used for DENSE analysis, we retrospectively analyzed a large number of DENSE studies that had been previously processed at our institution. To eliminate errors introduced by sources other than our simplification, we modified the original manual contours directly to create simplified boundaries rather than having someone manually perform the simple analysis as this would add inter-observer variation to the differences seen between the simple and original methods.

We proposed that the user can draw three contours (endocardial on end systole and diastole and epicardial on end diastole) to derive cardiac mechanics. These contours were selected because they are the three contours needed to derive LV ejection fraction, mass, and volumes. These three contours were used as follows:

1. The end diastolic endocardial and epicardial contours (“Resting Configuration”) were used to initialize the mesh (Figure 1C) for strain calculations (Figure 2, Column 1).

**Figure 2:**
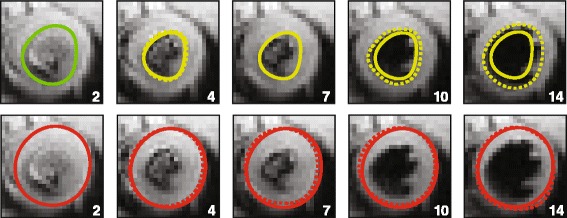
**Simplified contour generation.** Simplified contours (Solid Lines) were generated from the original user-defined contours (Dashed Lines) for end-diastolic and end-systolic images. The epicardial contour (red) was copied from the end-diastolic frame to all other images. The end-systolic endocardial boundary (yellow) was copied to all other images with the exception of the resting configuration (green). Frame numbers are given in the bottom right of each image.
2. The epicardial contour from the resting configuration (end diastole) was copied to all other frames in the cardiac cycle as it was assumed to represent the most radial extent of the myocardium throughout the cardiac cycle (Figure 2, Solid Red Line).
3. The endocardial contour from the end systolic image (defined by the smallest left ventricular volume) was copied to all other images in the series except for the reference frame (Figure 2, Green Line). This boundary theoretically represents the most central extent of the myocardium throughout the cardiac cycle (Figure 2, Solid Yellow Line).

A similar simplification was performed on the long-axis images. Cardiac strains and torsion were calculated exactly as described in the previous section except that the simplified contours were substituted for the original contours. The same parameters used for the initial processing of each dataset were maintained for the comparative analysis including the number of segments, location of phase unwrapping seed points, and location of the anterior insertion of the right ventricle. This consistency allowed for the direct comparison between the resulting strains and torsions derived during post-processing from 1) the original contours defined by the user on all image frames and 2) the three “simplified” contours.

### Comparison of peak strains and torsion

We compared the peak circumferential, radial, and longitudinal strains between the simplified and original contours using Bland-Altman analysis [16] and a modified coefficient of variation (CoV) (Equation 1). (*N* is the number of strain or torsion values, and *x*_o_ and *x*_*s*_ are the strains/torsions for the original and simplified contours, respectively)

1 $$ CoV\left({x}_o,{x}_s\right) = \frac{{\displaystyle {\sum}_{i=1}^N} St.Dev\left({x}_o\left[i\right],\ {x}_s\left[i\right]\right)}{\left|{\displaystyle {\sum}_{i=1}^N}\left({x}_o\left[i\right] + {x}_s\left[i\right]\right)\ /\ 2\right|} $$

### Comparison of strain and torsion curves

Although the agreement between peak strains is important, it may not be representative of the agreement between the shape of two strain curves since the values can disagree at any point other than the peak and there will be no effect on the computed peak strains. To account for this and fully characterize the error in the strain curves, we computed the root mean squared error (RMSE) (Equation 2) between all segmental strain curves.

2 $$ RMSE\left({x}_o,{x}_s\right) = \sqrt{\frac{{\displaystyle {\sum}_{i=1}^N}{\left({X}_o\left[i\right]-{X}_s\left[i\right]\right)}^2}{N}} $$

### Inter-observer agreement

We hypothesized that providing only three contours for DENSE analysis would result in accurate calculations of cardiac mechanics. To provide some context for “accurate”, we compared the differences to inter-observer variability. We computed the RMSE, CoV, and limits of agreement between two observers’ analyses of the same images using the original contouring method. Inter-observer reproducibility was also computed using our new simplified approach. Inter-observer agreement for torsion and radial and circumferential strain was assessed in 46 of the human short-axis imaging slices and a subset of 57 short-axis imaging slices from the mouse data. Inter-observer reproducibility of longitudinal strain was assessed in 28 of the human long-axis imaging slices and a subset of 36 long-axis imaging slices from the mouse data. The statistical significance of the differences in RMSE between the simple and inter-observer data was determined using a Student’s t-test.

### Comparison with other semi-automated techniques

A number of semi-automated techniques have been proposed to improve the efficiency of DENSE image analysis. Spottiswoode et al. developed a Motion-Guided Segmentation (MGS) method in which the measured displacements are used to propagate the contours from an end diastolic reference frame to all other frames over the cardiac cycle [13]. More recently, Gilliam et al. proposed an automated method for DENSE analysis which attempts to segment the myocardium by assessing the quality of the phase data [17]. We compared the accuracy of our simplified analysis to both of these methods by comparing the Bland Altman 95% limits of agreement [16]. Due to the fact that the original MGS manuscript only compared pixel-wise segmentation results, we performed MGS on our data to compare the resulting LV myocardial strains directly to the original manual contours.

## Results

### Comparison of peak strains and torsion

**Figure 3:**
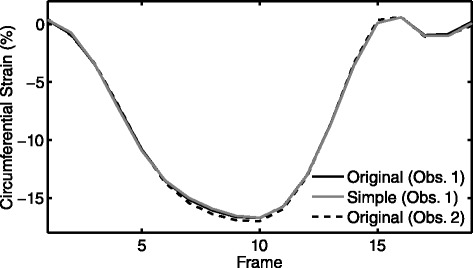
**Representative circumferential strain curves.** Representative circumferential strain curves for a mid-ventricular short-axis image are shown for the original analysis method, our simplified analysis method, and a separate observer’s analysis using the original method.

**Figure 4:**
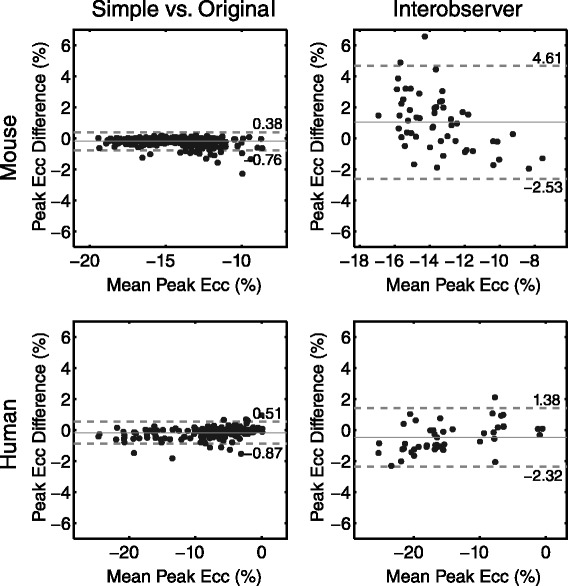
**Bland Altman limits of agreement for peak circumferential strain.** Simplified analysis resulted in very tight agreement of peak circumferential strain (Ecc) values compared to the original contours in mice (top left) and humans (bottom left). This agreement is markedly better than the corresponding inter-observer variability in peak circumferential strain values using the original analysis (right). Note that all strain values are in units of absolute strain i.e. they are *not* normalized.

We observed strong agreement between the strains computed from the simplified contours compared to the original contours (Figure 3). In particular, peak circumferential strain showed high accuracy for the simplified approach. The Bland Altman limits of agreement for peak circumferential strain were much tighter (Figure 4, left) than the inter-observer variability based on the original analysis (Figure 4, right) in both the mouse and human scans (Table 1).

**Table 1:** **Bland Altman limits of agreement for peak strains and torsions**

	**Mouse**		**Human**	
	**Simple**	**Interobserver**	**Simple**	**Interobserver**
Radial Strain (%)	−0.5 ± 4.0	−5.5 ± 13.1	−1.6 ± 4.2	3.3 ± 12.9
Circumferentaial strain (%)	−0.2 ± 0.6	1.0 ± 3.6	−0.2 ± 0.7	−0.5 ± 1.8
Longitudinal strain (%)	−0.5 ± 1.6	−0.2 ± 6.4	0.3 ± 2.9	0.4 ± 3.0
Torsion (deg/cm)	0.0 ± 0.8	0.3 ± 2.2	0.0 ± 0.3	−0.0 ± 0.4

Peak strain Bland Altman analysis.

In addition to peak circumferential strains, our simplified approach yielded accurate measures of radial and longitudinal strain, as well as torsion (Table 1). Our approach exhibited the most error when computing radial strain (Mouse: −0.5 ± 4.0%, Human: −1.6 ± 4.2%); however, it was still better than the inter-observer variability of the original analysis (Mouse: −5.5 ± 13.1%, Human: 3.3 ± 12.9%).

**Figure 5:**
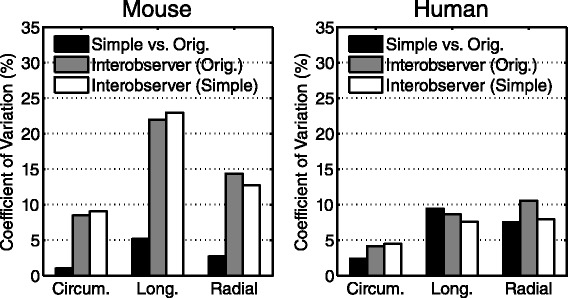
**Coefficient of variation between peak strain values.** There was superior agreement between the simplified and original analysis (black bars) compared to the inter-observer agreement from the original analysis (gray bars). The inter-observer agreement of the simplified analysis (white bars) was comparable to that of the original analysis in mice (left) and humans (right).

The simplified approach yielded superior coefficients of variation (CoVs) compared to the inter-observer CoVs in mice for circumferential (1.0 vs. 8.5%), longitudinal (5.2 vs. 21.9%), and radial strain (2.7 vs. 14.4%). In humans, the simple method had smaller CoVs compared to the inter-observer CoVs for circumferential (2.4 vs. 4.2%), and radial strain (7.5 vs. 10.6%) and a larger CoV for longitudinal strain (9.4 vs. 8.6%) (Figure 5).

### Comparison of strain and torsion curves

**Figure 6:**
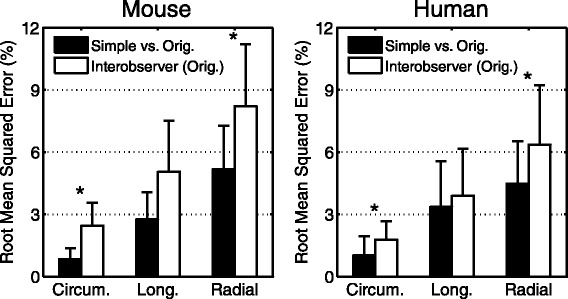
**Root mean squared error between strain values.** There was superior agreement between the simplified and original analysis (black bars) compared to the inter-observer agreement from the original analysis (white bars) in both mice (left) and humans (right). (*indicates p <0.001).

Not only did the simplified post-processing result in accurate peak strain values, but the accuracy was maintained throughout the entire cardiac cycle. The root mean squared error (RMSE) of the strain curves was comparable to the RMSE from the original inter-observer analysis (Figure 6).

### Comparison with other semi-automated techniques

**Figure 7:**
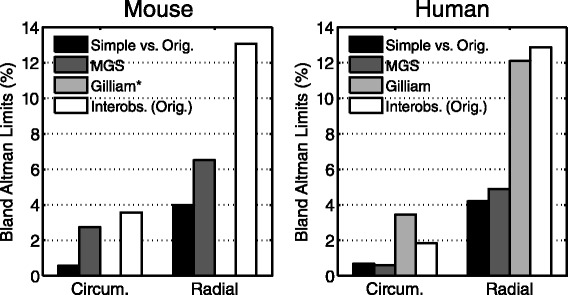
**Comparison with semi-** **automated techniques.** The agreement between the simplified and original analysis (black bars) was superior to both inter-observer error from the original analysis (white bars) and other automated methods (gray bars) in both mice (left) and humans (right). (*Gilliam data was obtained from the original manuscript [17] where data was only provided for human subjects).

The accuracy of the simplified analysis was equivalent or superior to both the accuracy of MGS and Gilliam’s automated approach (Figure 7).

### Effect of simplified analysis on inter-observer reproducibility

The simplified analysis of DENSE data had inter-observer reproducibility comparable to that of the original method. In mice, the coefficients of variation were 12.7%, 9.0%, and 23.0% for radial, circumferential, and longitudinal strain, respectively. In humans, the coefficients of variation were 7.9%, 4.5%, and 7.6% for radial, circumferential, and longitudinal strain, respectively (Figure 5).

### Effect on processing time

On average, 24 cardiac phases were acquired for each imaging slice, requiring 48 endocardial or epicardial contours to be drawn using the original method. The simplified approach only requires three contours per imaging slice resulting in a theoretical 94% improvement in efficiency. In our experience, manual segmentation of an entire imaging study (both short-axis and long-axis images) takes 45 minutes for human data and 20 minutes for mouse data. Using our simplified method, this time can be decreased to 3 and 1.2 minutes, respectively.

## Discussion

DENSE is an advanced CMR technique which allows for accurate, non-invasive assessment of cardiac mechanics. Such a technique has potential clinical utility; however, the amount of post-processing currently required may limit the clinical utilization of the technique. In the typical DENSE image acquisition, approximately 20 frames are acquired prospectively over the cardiac cycle, requiring the user to manually delineate 40 contours per imaging slice (an epicardial and endocardial contour on each image frame).

This study introduces and validates a radical approach to DENSE analysis in which only *three* contours are drawn on end diastolic and systolic images. We found that, using this approach, we could accurately compute cardiac mechanics using 94% less time. These results dramatically improve the clinical feasibility of DENSE.

### Comparison with inter-observer reproducibility

To provide some context, we directly compared the accuracy of strains and torsion computed from the simplified approach to the inter-observer variability. Overall we found that the error introduced due to the simplified approach was much less than the inter-observer variability for calculation of peak strains and torsion. Additionally, we found the inter-observer reproducibility of the simplified approach to be comparable to the original inter-observer variability. This indicates that the majority of the variation in DENSE-derived mechanics originates from the user-defined contour on the end diastolic reference frame. Although the contours throughout the rest of the cardiac cycle should be reasonable, their accuracy has little effect on the computed strains.

These results held true in data from both healthy and diseased mice and humans that were acquired across two institutions. Despite the tight Bland-Altman limits of the simple approach, the CoV was slightly higher than the inter-observer CoV for longitudinal strain in the human scans, but this difference is likely insignificant.

The agreement seen in the circumferential strain calculations is likely due to the large number of pixels available to compute circumferential strain. As such, a single erroneous pixel value (caused by a bad contour) is outweighed by the large number of correct pixels. In contrast, there are typically only 2–3 pixels across the myocardial wall that can be used to derive radial strains; therefore, the effect of an erroneous pixel is amplified. However, it has been demonstrated that the reproducibility of radial strain is impaired at the current spatial resolution, especially when considering subendocardial or subepicardial strain values [7,18]. The simplified analysis still introduced less error in radial strain than the inter-observer agreement for calculating radial strains. Similar to circumferential strain, the cardiac twist of an imaging slice was relatively immune to errors due to the large amount of data. As such, the torsion values exhibited agreement comparable to inter-observer analysis.

### Clinical implications

We anticipate that this simplification of the analysis of DENSE images will help facilitate more widespread adoption of this advanced cardiac imaging technique. Using the method introduced in this study, DENSE post-processing would only require the user to define three contours per imaging slice. This results in an estimated decrease of 94% in the post-processing time. The real efficiency may be even higher due to the fact that the end-systolic frame on which the user must delineate the endocardial boundary has excellent contrast between the blood pool and myocardium. Furthermore, the time required to segment an imaging slice is independent of the number of time frames acquired. This implies that high temporal resolution scans can be performed without prolonging the time required for post-processing. Processing time could potentially be reduced even further by removing the need for the user to manually select phase unwrapping seed points by employing phase quality-based unwrapping algorithms [14].

Haggerty et al. have demonstrated that the black-blood magnitude images generated from the DENSE acquisition can be used to accurately assess myocardial mass, ventricular volumes, and ejection fraction in mice [8]. It is important to note that the three contours required to perform robust approximation of mechanics are the *same* contours that one would need to compute ventricular volumes, mass and ejection fraction. This increases the clinical utility of DENSE because using data from one acquisition, three boundaries can be delineated to potentially compute both standard (ejection fraction, volumes and mass) and advanced (strains and torsion) measures of cardiac function. However, using DENSE magnitude images to quantify myocardial mass, ventricular volumes and ejection fraction has *not* yet been validated in humans, which is a logical next step.

### Comparison to segmentation techniques

In an attempt to reduce the post-processing time of large datasets, hundreds of segmentation methods and techniques have been developed [10-13]. For DENSE specifically, Spottiswoode, et al. developed an algorithm for segmenting the myocardium by using a set of two contours (endocardial and epicardial) combined with the displacement information encoded into the phase images [13]. The accuracy of their technique was assessed by directly comparing the resulting contours to manually-defined contours. They did not, however, compare the resulting strain or torsion values between the techniques. We performed motion guided segmentation on our data and found that the simplified approach had superior agreement with the original contours.

Similarly, we found that the agreement between the original analysis and the simplified approach was better than the agreement reported for Gilliam’s automated DENSE image analysis [17]. We suspect that this difference is partially due to the inability of the automated segmentation, which is based upon phase data, to perfectly discriminate between the myocardium, papillary muscles, and other stationary tissue.

### Limitations

In this study, all processing of the simplified contours was automated due to the large number of datasets. As a result, phase unwrapping errors could have been present in the simulated data derived from the simplified contours. However, if a user were to perform the proposed analysis themselves, proper phase unwrapping and therefore more accurate displacement information would be ensured. As such, the results presented here are essentially the worst-case scenario for the proposed approach. Additionally, our approach could be combined with automated phase-unwrapping techniques to further simplify DENSE post-processing [17].

In addition to the contour-based strain analysis presented in this manuscript, there are pixel-based approaches that are much less dependent upon user-drawn contours and can often compute mechanics with limited user input [19,20]. The simple method presented is specific to the contour-based methods.

We observed a minimal effect of simplified contours in our heterogeneous patient and mouse population. It would be beneficial to include more patients with a variety of cardiovascular diagnoses to further evaluate our results. We believe, however, that due to the strong agreement observed in the current population, the results are likely generalizable.

The theoretical efficiency presented in this manuscript was based purely on the reduction in the number of required contours. This number does not take into account the fixed amount of time required for the user to manually select the location of the anterior insertion of the right ventricle or the manual seed points to aid in the path-guided phase unwrapping. These steps, however, make up a small percentage of the post-processing time relative to the contouring of the myocardium therefore their effect will be minimal.

### Future implications

One of the added benefits of the simplified DENSE analysis is that the time required to contour the data is fixed for a given slice, therefore improving the feasibility of processing higher temporal resolution data. Although the temporal resolution of cine DENSE imaging is currently limited to approximately 16 milliseconds, most groups currently only obtain data with a temporal resolution of 32 milliseconds due to longer acquisition times and the prohibitive processing time required. High temporal resolution DENSE data may allow us to more accurately measure cardiac strain *rates* in addition to strains. Strain rates are theoretically less load-dependent than strains, and therefore may represent better measures of cardiac function [21].

Several studies have shown that fully three-dimensional cardiac mechanics can be derived using DENSE [22,23]. These full 3D acquisitions, while providing more data, also require additional post-processing time due to the increased number of imaging slices. This simplified processing could potentially be applied to three-dimensional analysis to make the post-processing more manageable and clinically viable.

In addition to DENSE, there are a number of CMR-based techniques for measuring cardiac mechanics and function [1-3,24]. Future studies could explore the sensitivity of these methods to the accuracy of the myocardial segmentation. We suspect that methods which rely directly upon the user-defined segmentation (regional wall motion and wall thickness) will be extremely sensitive to errors in the contours [24,25]. The effect, however, on phase-based analyses such as strain encoding and tissue velocity mapping should be explored in an effort to improve their clinical utility.

## Conclusions

The delineation of endocardial and epicardial boundaries is an essential post-processing step for deriving cardiac mechanics from DENSE CMR data. As with many advanced imaging techniques, clinical adoption of the technology requires minimization of the post-processing time. We have proposed a simplified processing technique for DENSE imaging which accurately measures cardiac mechanics 94% faster than existing techniques. By drastically simplifying post-processing, this technique moves DENSE assessment of cardiac function one step closer to clinical feasibility.
